# Domain-specific motor skill outcomes following a structured play-based motor intervention in preschool children: a cluster-randomized controlled trial

**DOI:** 10.3389/fspor.2026.1917249

**Published:** 2026-08-24

**Authors:** Massimiliano Agnese, Simone Massaro, Roberta Cottone, Antonino Scardina, Floreana Lo Dico, Samuel Honório, Marianna Bellafiore, Garden Tabacchi

**Affiliations:** 1 Sport and Exercise Sciences Research Unit, Department of Psychology, Educational Science and Human Movement, University of Palermo, Palermo, Italy; 2 Department of Neurosciences, Biomedicine and Movement Sciences, University of Verona, Verona, Italy; 3 Sport Physical Activity and Health Research & Innovation Center – SPRINT-IPCB, Polytechnic University of Castelo Branco, Castelo Branco, Portugal; 4 Insight: Piaget Research Center for Ecological Human Development, Piaget Institute, Almada, Portugal

**Keywords:** cluster-randomized controlled trial, fundamental motor skills, gross motor development, motor competence, physical literacy, play-based intervention, preschool children, TGMD–3

## Abstract

**Background:**

Fundamental motor skills (FMS) are key components of early motor competence and part of the physical competence domain of physical literacy. However, less is known about domain-specific effects of structured play-based motor programs in preschool children and whether these effects vary by baseline motor competence and age.

**Materials and methods:**

This cluster-randomized controlled trial included 160 children aged 3–6 years from eight preschool classes, with classes randomly allocated to an intervention group (IG; five classes, *n* = 98) or a control group (CG; three classes, *n* = 62). The IG received an 8-week structured play-based motor intervention twice weekly, whereas the CG continued usual preschool activities. FMS were assessed pre- and post-intervention using the Test of Gross Motor Development–Third Edition. Primary outcomes were locomotor raw scores, ball skills raw scores, and the Gross Motor Index (GMI). Primary complete-case analyses included 139 children and used ANCOVA models with post-intervention scores as dependent variables, group and sex as fixed factors, and baseline score and age as covariates. Main effects and interactions were tested using *F*-tests, with partial eta squared (*η*^2^_p_) reported. Mixed-effects models with preschool class as a random intercept and multiple imputation for missing post-intervention data were conducted as sensitivity analyses. The study was registered at ClinicalTrials.gov (NCT07624695; https://clinicaltrials.gov/study/NCT07624695).

**Results:**

Compared with the CG, the IG showed significantly higher post-intervention locomotor scores, ball skills scores, and GMI. Group effects were significant for locomotor skills, *F*(1,131) = 31.37, *p* < .001, *η*^2^_p_ = .193; ball skills, *F*(1,131) = 94.81, *p* < .001, *η*^2^_p_ = .420; and GMI, *F*(1,131) = 73.24, *p* < .001, *η*^2^_p_ = .359. Significant group × baseline score interactions were observed for all outcomes, while group × age interactions were significant for ball skills and GMI. Sensitivity analyses yielded results consistent with the complete-case findings.

**Conclusions:**

Among preschool children, a structured play-based motor intervention was associated with greater improvements in FMS than usual preschool activities. Findings suggest that intervention effects may vary by motor domain, baseline competence, and age, supporting differentiated and developmentally responsive approaches to early childhood motor skill development.

## 1 Introduction

Early childhood represents a key developmental period during which movement experiences and play contribute to cognitive, physical, motor, social, and emotional development and may help establish lifelong health-related behaviors (1). During this period, active play provides important opportunities for experiential learning and interaction with the surrounding environment (2). Fundamental motor skills (FMS) are a central component of early motor competence and represent the basic movement patterns underlying more complex and specialized motor behaviors. FMS are generally categorized into locomotor skills, which require body movement through space, and object-control skills, which involve the manipulation of objects (3). Proficiency in these skills has been consistently associated with higher levels of physical activity participation, physical fitness, and more favorable health-related outcomes during childhood and adolescence (4).

Despite the recognized importance of physical activity and motor competence in early childhood, excess body weight and insufficient movement behaviors remain important public health concerns. According to the World Health Organization (WHO) European Childhood Obesity Surveillance Initiative, 25% of children aged 7–9 years are living with overweight, including obesity, supporting the relevance of preventive strategies beginning early in childhood (5). Evidence on adherence to physical activity recommendations among preschool-aged children remains variable across studies, partly because of differences in accelerometer-based measurement methods and cut-points (6). In parallel, many children demonstrate insufficient levels of fundamental motor skill proficiency, which may negatively influence participation in physical activity (7). Poor proficiency in these skills during the preschool years has been associated with increased risk of obesity and chronic diseases later in life (8).

In this context, movement-related competencies play a central role in shaping early health behaviors. Among these, physical literacy (PL) provides a broader framework for understanding children's engagement with movement and physical activity, particularly in early childhood. From a holistic perspective, PL encompasses the capacity to interact effectively with the environment through embodied movement, integrating physical competence with perception, experience, anticipation, and appropriate responses to movement-related situations (9). At the same time, PL is recognized as a multidimensional construct encompassing cognitive, affective, behavioral, and physical domains (10). In the present study, FMS and motor competence are therefore considered indicators of the physical competence domain of PL, rather than measures of PL as a multidimensional construct.

Importantly, locomotor and object-control skills may show different developmental patterns, supporting the relevance of examining domain-specific intervention effects. Evidence from intervention studies indicates that structured, developmentally appropriate motor programs can enhance FMS in early childhood, with improvements observed in both locomotor and object-control skills, including in preschool-based physical education programs (11, 12). Although unstructured free play provides valuable opportunities for spontaneous movement, exploration, and social interaction, it may not consistently ensure the repeated task-specific practice, progressive difficulty, instructional feedback, and guided refinement required to improve specific motor skills. In contrast, structured play-based interventions can preserve the motivational and developmentally appropriate features of play while embedding planned motor tasks, progression, and adult guidance (8, 13). These elements may be particularly relevant for object-control skills, which involve object manipulation and may require structured, supervised, and task-specific practice to improve more complex coordinative patterns such as dribbling and catching (14).

Despite these advances, some aspects of motor skill development in early childhood remain incompletely understood. In particular, greater attention is still needed to domain-specific intervention effects, as well as to the potential influence of individual factors such as baseline motor competence and age on intervention outcomes. Clarifying these aspects in school-based settings may contribute to a more nuanced understanding of how FMS develop in response to structured programs.

Against this background, the present study was conducted as part of the Pre-HELP (Preschoolers’ Health Literacy Project), a multidisciplinary initiative aimed at promoting health-related competencies in preschool children through integrated educational strategies. Health literacy is increasingly conceptualized as encompassing the cognitive and social skills required to access, understand, and use health-related information in ways that promote and maintain health across the lifespan (15). In preschool children, gross motor development has also been associated with emergent literacy and other health-related educational domains, supporting the relevance of integrated approaches to early childhood development (16). Within the broader Pre-HELP framework, the present paper focuses specifically on motor competence, with particular attention to FMS development.

The primary aim of this study was to evaluate the effects of a structured play-based motor intervention on FMS development in preschool children, compared with usual preschool activities. Secondary aims were to examine domain-specific outcomes across locomotor and object-control skills and to explore whether intervention effects varied according to baseline motor competence and age. It was hypothesized that children participating in the intervention would show greater improvements in FMS and overall motor competence than children continuing usual preschool activities, with intervention effects potentially differing across motor domains and participant characteristics.

## 2 Materials and methods

### 2.1 Study design and participants

A cluster-randomized controlled trial (cRCT) was conducted with a sample of 160 preschool-aged children (3–6 years). Two public schools in the municipality of Palermo were recruited based on institutional availability and willingness to participate. Within each school, eligible preschool classes were identified, and participating classes were selected through a random draw conducted by the school staff. Preschool classes served as the units of randomization. A total of eight preschool classes were included and randomly allocated to either the intervention group (IG; five classes, *n* = 98 children) or the control group (CG; three classes, *n* = 62 children). Allocation was performed using a computer-generated random sequence by an independent researcher who was not involved in data collection. Cluster randomization at the preschool-class level was adopted to preserve the natural classroom setting and minimize contamination between participants.

Inclusion criteria were: (a) enrollment in a participating preschool class, (b) age between 3 and 6 years, and (c) regular participation in usual preschool activities. No exclusion criteria were defined.

Before the intervention, a power analysis was conducted using G*Power to estimate the required sample size for detecting a medium effect size (*f* = 0.25) in an analysis of covariance (ANCOVA) model with two groups and two covariates, assuming an alpha level of.05 and statistical power of.80. The analysis indicated a required total sample size of 128 participants. The final sample size of 160 children exceeded this estimate. However, the calculation was based on an individual-level ANCOVA model and did not explicitly account for the cluster-randomized design or the potential design effect. Therefore, the power analysis should be interpreted as an approximate estimate rather than a definitive cluster-adjusted sample size calculation.

Prior to baseline assessment, children were familiarized with the testing environment during anthropometric assessment and through standardized explanation and demonstration of the motor tasks. This familiarization procedure was intended to facilitate task comprehension and reduce potential test-related anxiety. Baseline data were collected before the intervention period began. During the intervention period, children in the IG received the structured play-based motor intervention, whereas children allocated to the CG continued their usual preschool activities throughout the study period. Usual activities consisted of ordinary educational and play routines provided by the preschool and could include teacher-supervised spontaneous play as well as classroom or outdoor activities. No additional structured motor program was provided to the CG as part of the study. Post-intervention assessments were conducted for both groups following the 8-week intervention period. Post-intervention data were unavailable for 6 children in the IG and 15 children in the CG, primarily due to reduced attendance toward the end of the preschool year. As reported in the statistical analysis section, primary inferential analyses were conducted on complete cases only.

The study was conducted between January and June 2025. Following a preparatory phase, baseline assessments and intervention delivery were carried out in the participating schools between March and June.

Because the motor program was delivered as a visible class-level activity in the school setting, participants and intervention staff could not be blinded to group allocation. Outcome assessors were not blinded to group allocation. Given the pragmatic school-based implementation of the broader project, the preschool age of the participants, and the need for repeated coordination with teachers and school staff for scheduling and supervision, assessor blinding was not feasible throughout the assessment process. Motor assessments were conducted on school premises using the same procedures at baseline and post-intervention. To mitigate potential detection bias, each child was assessed individually using standardized administration procedures, predefined task demonstrations, identical verbal instructions, and criterion-based scoring across time points.

Written informed consent was obtained from the parents or legal guardians of all participating children. The study was approved by the Bioethics Committee of the University of Palermo (Approval No. 269/2024) and conducted in accordance with the Declaration of Helsinki. The study was retrospectively registered on ClinicalTrials.gov (Identifier: NCT07624695), as prospective registration had not been finalized before baseline data collection. Participation was voluntary, and parents were informed of their right to withdraw their child from the study at any time without consequences.

### 2.2 Description of the intervention

The intervention was designed as a structured play-based motor program aimed at promoting FMS in preschool-aged children. A play-based approach served as the core pedagogical principle guiding the design and delivery of all activities. The program lasted 8 weeks and was delivered twice per week, with each session lasting approximately 60 min.

Each session followed a standardized structure consisting of a warm-up phase, a central phase focused on FMS development, and a cool-down phase. The central phase included activities targeting locomotor skills, dynamic balance, coordination, and object-control skills, delivered through playful and game-based tasks. The intervention was organized progressively, moving from the exploration of basic movement patterns toward consolidation and increased task complexity. Activities were adapted to children's age and motor competence, and a variety of equipment was used to promote engagement, task variability, and diversified movement experiences.

Intervention sessions were delivered by two professionals trained in motor development and experienced in the implementation of structured movement programs for young children. Sessions took place in indoor school gymnasiums or designated physical activity spaces within the preschool facilities, ensuring adequate space and safety conditions for the execution of all tasks. To enhance intervention consistency, sessions followed a predefined structure and were delivered according to a common program framework shared by the two instructors. Written attendance records were kept for each session. Among IG children with post-intervention data, 87 of 92 children (94.6%) attended at least 9 of the 16 intervention sessions. Attendance data were used descriptively and were not applied as an exclusion criterion for the analyses. However, no independent fidelity checklist or formal quantitative assessment of intervention fidelity was implemented.

### 2.3 Outcome measures

Anthropometric measurements were obtained at baseline using standardized procedures. Body weight was measured using a digital scale, and standing height was measured using a portable stadiometer. Measurements were performed twice for each child, and the mean value was used for analysis. Children were assessed barefoot and wearing light clothing. Body mass index (BMI) was calculated as weight in kilograms divided by height in meters squared (kg/m^2^).

BMI values were categorized according to WHO age- and sex-specific BMI-for-age cut-offs. For children aged 36–60 months, classification was based on the WHO Child Growth Standards (17), whereas for children aged 61–72 months it was based on the WHO Growth Reference for school-aged children and adolescents (18). Children were classified as underweight, normal weight, overweight, or obese. BMI categories were used for descriptive purposes only.

FMS were assessed using the Test of Gross Motor Development–Third Edition (TGMD–3), a standardized, process-oriented instrument designed to assess gross motor skill performance in children aged 3–10 years (19). The TGMD–3 evaluates 13 skills across two domains: six locomotor skills and seven ball skills, the latter representing the object-control domain of the test. Each skill is evaluated according to specific performance criteria reflecting the quality of movement execution.

Children performed two scored trials following standardized instructions and demonstration by the examiner. Given the young age of the participants, one practice trial was allowed to ensure adequate task comprehension. Each performance criterion was scored dichotomously (1 = criterion present; 0 = criterion absent), and raw scores were obtained by summing the criteria across trials. The maximum raw score is 46 for locomotor skills and 54 for ball skills.

Raw scores can be converted into age- and sex-adjusted standard scores and combined to obtain the Gross Motor Index (GMI), a norm-referenced composite score (M = 100, SD = 15) representing overall motor competence. In the present study, locomotor and ball skills raw scores were used in the primary analyses to ensure greater sensitivity to intervention-related changes over time compared to standard scores.

Assessments were conducted at both time points by the same trained personnel following standardized administration procedures to ensure consistency in data collection. Each child was assessed individually. Testing took place in indoor school gymnasiums or designated activity rooms, providing sufficient space for the administration of all tasks in accordance with TGMD–3 manual guidelines. Prior to data collection, assessors were familiarized with the test scoring procedures to promote scoring consistency across testing sessions.

### 2.4 Statistical analysis

Descriptive statistics are reported as mean ± standard deviation (SD) for continuous variables and as frequencies and percentages for categorical variables. Baseline differences between groups were examined using independent-samples t-tests for continuous variables and chi-square tests or Fisher's exact test, as appropriate, for categorical variables. Primary inferential analyses were conducted on complete cases only, including participants with both baseline and post-intervention outcome data. Missing post-intervention data were not imputed for the primary complete-case analyses. Therefore, attrition analyses were performed to evaluate whether children without post-intervention data differed from completers in baseline demographic and motor competence characteristics. For continuous attrition analyses, Welch's correction was applied to account for potential unequal variances between completers and dropouts. Categorical attrition analyses were conducted using chi-square tests or Fisher's exact test, as appropriate.

As a sensitivity analysis for missing post-intervention data, multiple imputation was conducted for the three post-intervention outcomes. Thirty imputed datasets were generated using predictive mean matching within the multivariate imputation by chained equations framework (20). The imputation model included group, sex, age, baseline BMI, baseline locomotor score, baseline ball skills score, baseline GMI, and the available post-intervention outcome data. Baseline-adjusted ANCOVA models were then fitted across imputed datasets to estimate the main group effect for each outcome, and estimates were pooled according to Rubin's rules (21). Multiple imputation analyses were conducted in R (version 4.6.1) using the mice package (version 3.19.0).

Intervention effects were examined using separate ANCOVA models for post-intervention locomotor raw scores, ball skills raw scores, and GMI scores. In each model, group (IG vs. CG) and sex were included as fixed factors, whereas the corresponding baseline score and age were entered as covariates. Continuous predictors, namely age and baseline score, were mean-centered prior to analysis to reduce multicollinearity and improve coefficient interpretability.

To test potential moderation effects, interaction terms for group × age and group × sex were included in the models. In addition, the group × baseline score interaction was tested to examine potential violations of the homogeneity of regression slopes assumption and to assess whether intervention effects varied according to baseline motor competence. Significant interactions were further examined by estimating model-based marginal means and estimated group differences at representative values of the moderator, including the mean and ±1 standard deviation for continuous moderators. For significant interactions, estimated group differences at representative moderator values were reported with 95% confidence intervals (CIs).

To quantify potential clustering within preschool classes, intraclass correlation coefficients (ICCs) were estimated from baseline data using linear mixed-effects null models (22). Because the trial used cluster randomization at the preschool-class level, linear mixed-effects models including preschool class as a random intercept were also fitted as sensitivity analyses (23). These models used the same outcome variables, fixed factors, covariates, and interaction terms as the corresponding ANCOVA models, with the addition of preschool class as a random intercept to account for the nested data structure. Given the limited number of clusters, ANCOVA models were retained as the primary analytic approach, whereas mixed-effects models were interpreted as sensitivity analyses to examine whether the findings were robust after accounting for clustering by class (22, 23).

Model assumptions were assessed by visually inspecting residual distributions and Q-Q plots, complemented by Shapiro–Wilk tests for residual normality (24). Homogeneity of variances was evaluated using Levene's test (25). For the ball skills outcome, for which Levene's test indicated heterogeneity of variances, an additional heteroskedasticity-robust sensitivity analysis using heteroskedasticity-consistent type 3 (HC3) standard errors was conducted (26). In the presence of assumption violations, results were interpreted in conjunction with residual diagnostics and sensitivity analyses. Main effects and interactions were assessed using *F*-tests, and effect sizes were reported as partial eta squared (*η*^2^_p_). Statistical significance was set at *p* < .05.

For visualization purposes, a group-stratified regression plot was generated using GraphPad Prism (version 11.0.1) to illustrate the association between baseline and post-intervention GMI scores across groups. For descriptive purposes only, change scores (Δ = post-intervention−baseline) were calculated for locomotor and ball skills raw scores. Within the IG, Pearson correlation analyses were conducted to explore associations between age, baseline motor competence, and change scores, as well as between changes in locomotor and ball skills. Given their exploratory purpose, these analyses were interpreted descriptively. Descriptive statistics, baseline group comparisons, attrition analyses, primary complete-case ANCOVA models, estimated marginal means and estimated group differences, model assumption checks, ICC estimates, mixed-effects sensitivity analyses, and exploratory correlation analyses were performed using Jamovi (version 2.3.21.0). The HC3 robust sensitivity analysis was implemented through the Rj Editor module in Jamovi.

## 3 Results

**Figure 1:**
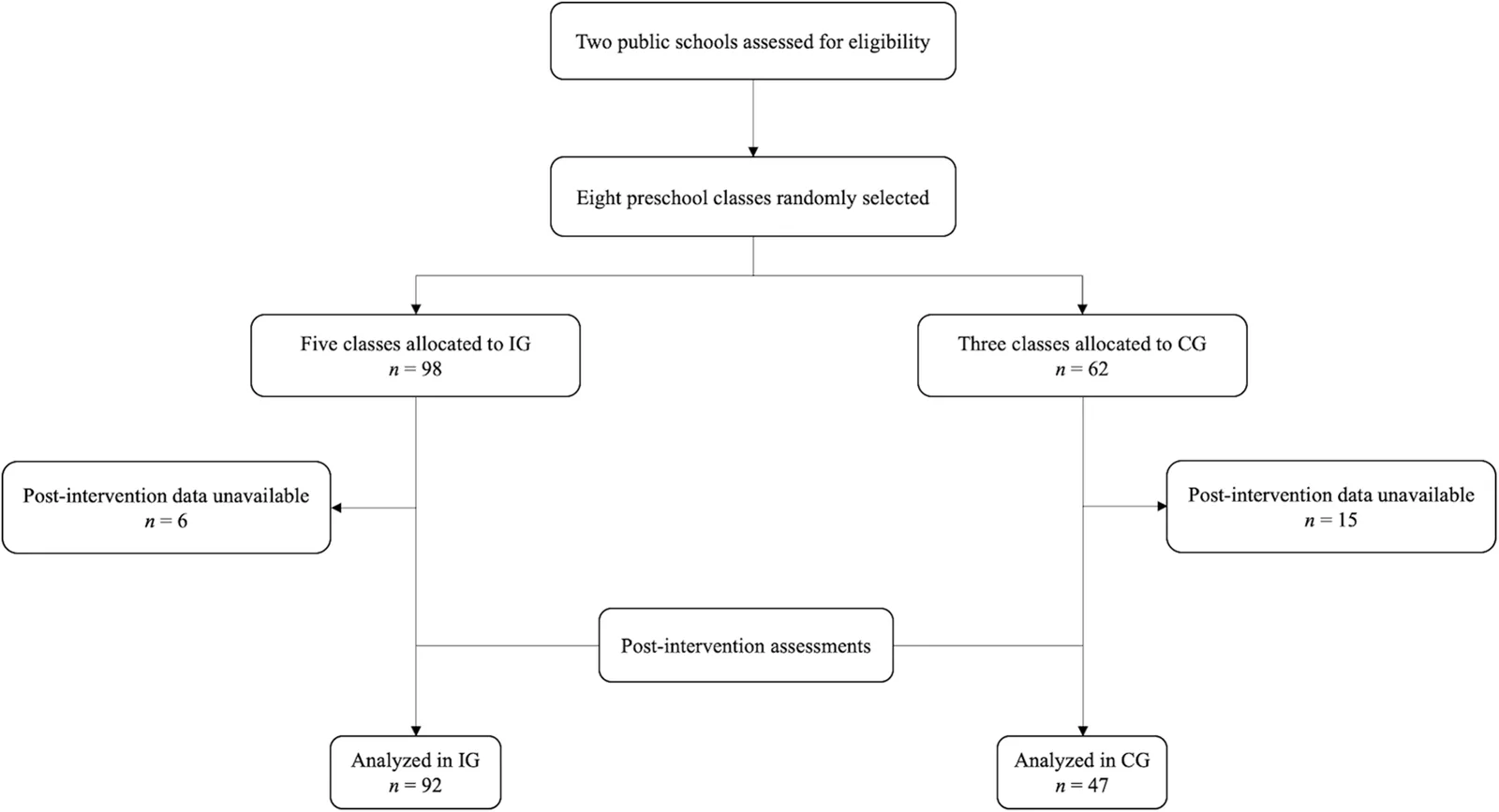
Flow diagram of school recruitment, cluster randomization, allocation, post-intervention assessment, and analysis.

At baseline, the sample included 160 children (IG = 98; CG = 62), with post-intervention data available for 139 participants (IG = 92; CG = 47). Attrition analyses showed no statistically significant differences between completers and dropouts in baseline age, anthropometric characteristics, sex distribution, BMI category, locomotor score, ball skills score, or GMI. Dropouts showed descriptively higher baseline ball skills scores than completers, although this difference did not reach statistical significance after Welch's correction for unequal variances (*p* = .061). Attrition differed significantly by group, with a higher dropout proportion in the CG (15/62, 24.2%) than in the IG (6/98, 6.1%; *p* < .001). The flow of participants through the study is presented in Figure 1, and the comparison between completers and dropouts is reported in Supplementary Table S1.

To further examine the robustness of the findings to missing post-intervention data, multiple imputation sensitivity analyses were conducted for the main intervention effects. Pooled estimates across 30 imputed datasets showed that the adjusted group effect remained statistically significant for locomotor scores (estimate = 6.92, 95% CI: 4.14–9.70, *p* < .001), ball skills scores (estimate = 13.05, 95% CI: 10.20–15.91, *p* < .001), and GMI (estimate = 17.21, 95% CI: 12.70–21.71, *p* < .001), yielding results consistent with the complete-case findings (Supplementary Table S2).

At baseline, the total sample had a mean age of 4.72 ± 0.85 years, body weight of 18.42 ± 3.04 kg, height of 1.07 ± 0.07 m, and BMI of 16.12 ± 1.53 kg/m^2^ (Table 1). The sample included 82 girls (51.2%) and 78 boys (48.8%). Most participants were classified as normal weight (73.8%), with 20.0% overweight and 6.3% obese (Table 2). Baseline characteristics were comparable between groups, with no statistically significant differences observed for age and anthropometric variables (independent-samples t-tests) or for sex distribution and BMI categories (chi-square tests and Fisher's exact test, respectively) (Tables 1, 2).

**Table 1:** Baseline age and anthropometric characteristics of the study sample.

Variable	Total sample (Mean ± SD)	IG (Mean ± SD)	CG (Mean ± SD)	*p*
Age (years)	4.72 ± 0.85	4.72 ± 0.85	4.71 ± 0.87	.918
Weight (kg)	18.42 ± 3.04	18.32 ± 3.13	18.57 ± 2.90	.615
Height (m)	1.07 ± 0.07	1.06 ± 0.06	1.07 ± 0.08	.489
BMI (kg/m^2^)	16.12 ± 1.53	16.11 ± 1.63	16.12 ± 1.37	.995

Values are presented as mean ± standard deviation. *p*-values refer to independent-samples t-tests comparing groups at baseline.

BMI, body mass index; CG, control group; IG, intervention group; SD, standard deviation.

**Table 2:** Baseline categorical characteristics of the study sample.

Variable	Total sample *n* (%)	IG *n* (%)	CG *n* (%)	*p*
Female	82 (51.2%)	52 (53.1%)	30 (48.4%)	.564
Male	78 (48.8%)	46 (46.9%)	32 (51.6%)	
Normal weight	118 (73.8%)	71 (72.4%)	47 (75.8%)	.485
Overweight	32 (20.0%)	19 (19.4%)	13 (21.0%)	
Obese	10 (6.3%)	8 (8.2%)	2 (3.2%)	

Values are presented as number (percentage). *p*-values refer to chi-square tests or Fisher's exact test, as appropriate, comparing distributions between groups.

CG, control group; IG, intervention group.

Descriptive statistics for baseline and post-intervention motor competence outcomes are presented in Table 3. No significant between-group differences were observed at baseline for locomotor scores, ball skills scores, or GMI.

**Table 3:** Motor competence outcomes at baseline and post-intervention.

Variable	Time	IG (Mean ± SD)	CG (Mean ± SD)	*p*
Locomotor score	PRE	17.46 ± 9.74	20.21 ± 10.06	.088
	POST	32.66 ± 10.94	26.89 ± 7.69	
Ball skills score	PRE	10.89 ± 4.08	12.08 ± 4.34	.081
	POST	29.09 ± 12.29	16.77 ± 7.67	
GMI	PRE	90.03 ± 13.61	93.34 ± 13.75	.138
	POST	116.53 ± 16.38	100.11 ± 10.85	

Values are presented as mean ± standard deviation. Locomotor and ball skills scores refer to raw scores. *p*-values refer to between-group differences at baseline (independent-samples t-tests).

CG, control group; GMI, Gross Motor Index; IG, intervention group; POST, post-intervention; PRE, baseline; SD, standard deviation.

ICCs estimated from baseline null models indicated that between-class differences accounted for 10.6% of the variance in locomotor scores, 7.8% in ball skills scores, and 9.9% in GMI (Table 4).

**Table 4:** Intraclass correlation coefficients (ICCs) for baseline outcomes.

Variable	ICC (%)
Locomotor score	10.6
Ball skills score	7.8
GMI	9.9

ICC values are expressed as percentages of total variance attributable to preschool-class level clustering at baseline. Locomotor and ball skills scores refer to raw scores.

GMI, Gross Motor Index; ICCs, intraclass correlation coefficients.

Results of the ANCOVA models are presented in Table 5. For locomotor post-intervention scores, ANCOVA revealed significant effects of group (*F*(1,131) = 31.37, *p* < .001, *η*^2^_p_ = .193), baseline score (*F*(1,131) = 55.07, *p* < .001, *η*^2^_p_ = .296), and age (*F*(1,131) = 7.64, *p* = .007, *η*^2^_p_ = .055), whereas sex was not significant (*p* = .988). A significant group × baseline interaction was observed (*F*(1,131) = 4.90, *p* = .029, *η*^2^_p_ = .036), while group × age and group × sex interactions were not significant. No substantial violations of model assumptions were detected.

**Table 5:** ANCOVA results for post-intervention motor competence scores.

Variable	Effect	*F*	*p*	*η* ^2^_p_
Locomotor score	Group	31.37	<.001	.193
	Baseline score	55.07	<.001	.296
	Age	7.64	.007	.055
	Sex	0.00	.988	<.001
	Group × Baseline score	4.90	.029	.036
	Group × Age	0.07	.797	.001
	Group × Sex	0.01	.939	<.001
Ball skills score	Group	94.81	<.001	.420
	Baseline score	14.34	<.001	.099
	Age	29.03	<.001	.181
	Sex	26.92	<.001	.170
	Group × Baseline score	6.66	.011	.048
	Group × Age	4.47	.036	.033
	Group × Sex	0.07	.791	.001
GMI	Group	73.24	<.001	.359
	Baseline score	37.11	<.001	.221
	Age	0.02	.896	<.001
	Sex	7.59	.007	.055
	Group × Baseline score	8.63	.004	.062
	Group × Age	11.38	<.001	.080
	Group × Sex	0.28	.595	.002

Post-intervention scores were entered as dependent variables. Locomotor and ball skills scores refer to raw scores. Models included group, centered baseline score, centered age, sex, group × baseline score, group × age, and group × sex interactions. F values are reported with *df* = 1,131 for all effects. *η*^2^_p_ = partial eta squared.

ANCOVA, analysis of covariance; GMI, Gross Motor Index.

For ball skills post-intervention scores, ANCOVA showed significant effects of group (*F*(1,131) = 94.81, *p* < .001, *η*^2^_p_ = .420), baseline score (*F*(1,131) = 14.34, *p* < .001, *η*^2^_p_ = .099), age (*F*(1,131) = 29.03, *p* < .001, *η*^2^_p_ = .181), and sex (*F*(1,131) = 26.92, *p* < .001, *η*^2^_p_ = .170). Significant group × baseline (*F*(1,131) = 6.66, *p* = .011, *η*^2^_p_ = .048) and group × age (*F*(1,131) = 4.47, *p* = .036, *η*^2^_p_ = .033) interactions were observed, whereas the group × sex interaction was not significant (*p* = .791). A violation of homogeneity of variances was detected (Levene's *p* = .017), while residuals were normally distributed. Therefore, a heteroskedasticity-robust sensitivity analysis using HC3 standard errors was conducted. In this analysis, the group effect remained statistically significant (*F*(1,131) = 106.32, *p* < .001). All main effects remained statistically significant, and the same pattern of significance was observed for the interaction terms: the group × baseline score interaction (*F*(1,131) = 4.71, *p* = .032) and the group × age interaction (*F*(1,131) = 4.51, *p* = .036) remained significant, whereas the group × sex interaction remained non-significant (*p* = .772).

For GMI post-intervention scores, ANCOVA revealed significant effects of group (*F*(1,131) = 73.24, *p* < .001, *η*^2^_p_ = .359), baseline score (*F*(1,131) = 37.11, *p* < .001, *η*^2^_p_ = .221), and sex (*F*(1,131) = 7.59, *p* = .007, *η*^2^_p_ = .055), whereas age was not significant (*p* = .896). Significant group × baseline (*F*(1,131) = 8.63, *p* = .004, *η*^2^_p_ = .062) and group × age (*F*(1,131) = 11.38, *p* < .001, *η*^2^_p_ = .080) interactions were observed, while the group × sex interaction was not significant. No substantial violations of model assumptions were detected (Table 5).

Because sex was significantly associated with ball skills and GMI, sex-specific descriptive statistics for these outcomes are provided in Supplementary Table S3. However, group × sex interactions were not significant for any outcome.

Sensitivity analyses based on linear mixed-effects models including preschool class as a random intercept yielded results consistent with the ANCOVA models in both direction and statistical significance. Post-intervention clustering effects were minimal for locomotor scores and GMI, while a small residual clustering effect was observed for ball skills (ICC ≈ 0.07).

To facilitate interpretation of significant interaction effects, estimated marginal means (EMMs) and estimated group differences derived from the mixed-effects models are presented in Table 6. For the locomotor outcome, the model-estimated difference between groups increased across baseline score levels, from approximately 4.0 points at low baseline values (−1 SD) to 10.6 points at high baseline values (+1 SD).

**Table 6:** Estimated marginal means (EMMs) for significant interaction effects.

Variable	Effect	Moderator level	IG Mean (SE)	CG Mean (SE)	Difference (95% CI)	*p*
Locomotor score	Group × Baseline score	−1 SD	26.35 (1.04)	22.27 (1.79)	4.08 (−0.02 to 8.17)	.051
		Mean	33.42 (0.73)	26.09 (1.05)	7.33 (4.79 to 9.87)	<.001
		+1 SD	40.50 (1.17)	29.92 (1.40)	10.58 (6.97 to 14.19)	<.001
Ball skills score	Group × Baseline score	−1 SD	24.79 (1.44)	15.20 (2.25)	9.59 (3.86 to 15.31)	.003
		Mean	29.94 (1.24)	16.47 (1.68)	13.47 (8.28 to 18.66)	<.001
		+1 SD	35.09 (1.54)	17.74 (2.01)	17.35 (11.75 to 22.95)	<.001
	Group × Age	−1 SD	24.47 (1.51)	14.28 (2.02)	10.19 (4.66 to 15.72)	.002
		Mean	29.94 (1.24)	16.47 (1.68)	13.47 (8.28 to 18.66)	<.001
		+1 SD	35.40 (1.48)	18.66 (2.05)	16.74 (11.24 to 22.25)	<.001
GMI	Group × Baseline score	−1 SD	107.53 (1.88)	95.83 (2.85)	11.70 (4.50 to 18.90)	.003
		Mean	117.63 (1.46)	99.37 (2.01)	18.26 (12.01 to 24.51)	<.001
		+1 SD	127.73 (2.01)	102.92 (2.51)	24.81 (17.80 to 31.83)	<.001
	Group × Age	−1 SD	113.79 (1.92)	102.86 (2.66)	10.93 (3.95 to 17.92)	.004
		Mean	117.63 (1.46)	99.37 (2.01)	18.26 (12.01 to 24.51)	<.001
		+1 SD	121.47 (1.93)	95.89 (2.62)	25.58 (18.66 to 32.50)	<.001

Estimated marginal means were derived from linear mixed-effects models. Values represent adjusted post-intervention scores at representative levels of the moderator (mean ± 1 SD), holding other covariates constant. Differences were calculated as IG−CG. Ninety-five percent confidence intervals and *p*-values refer to the estimated group differences at each moderator level, with lower and upper confidence limits shown in parentheses after each group difference. Standard errors (SEs) in parentheses refer to estimated marginal means. Locomotor and ball skills scores refer to raw scores.

CG, control group; CI, confidence interval; EMMs, estimated marginal means; GMI, Gross Motor Index; IG, intervention group; SD, standard deviation; SE, standard error.

For the ball skills outcome, greater between-group differences were estimated across increasing baseline score and age levels, ranging from approximately 9.6 points at low baseline values (−1 SD) to 17.4 points at high baseline values (+1 SD), and from 10.2 points at younger ages (−1 SD) to 16.7 points at older ages (+1 SD).

For GMI, a similar pattern was observed across increasing baseline score and age levels, ranging from approximately 11.7 points at low baseline values (−1 SD) to 24.8 points at high baseline values (+1 SD), and from 10.9 points at younger ages (−1 SD) to 25.6 points at older ages (+1 SD).

**Figure 2:**
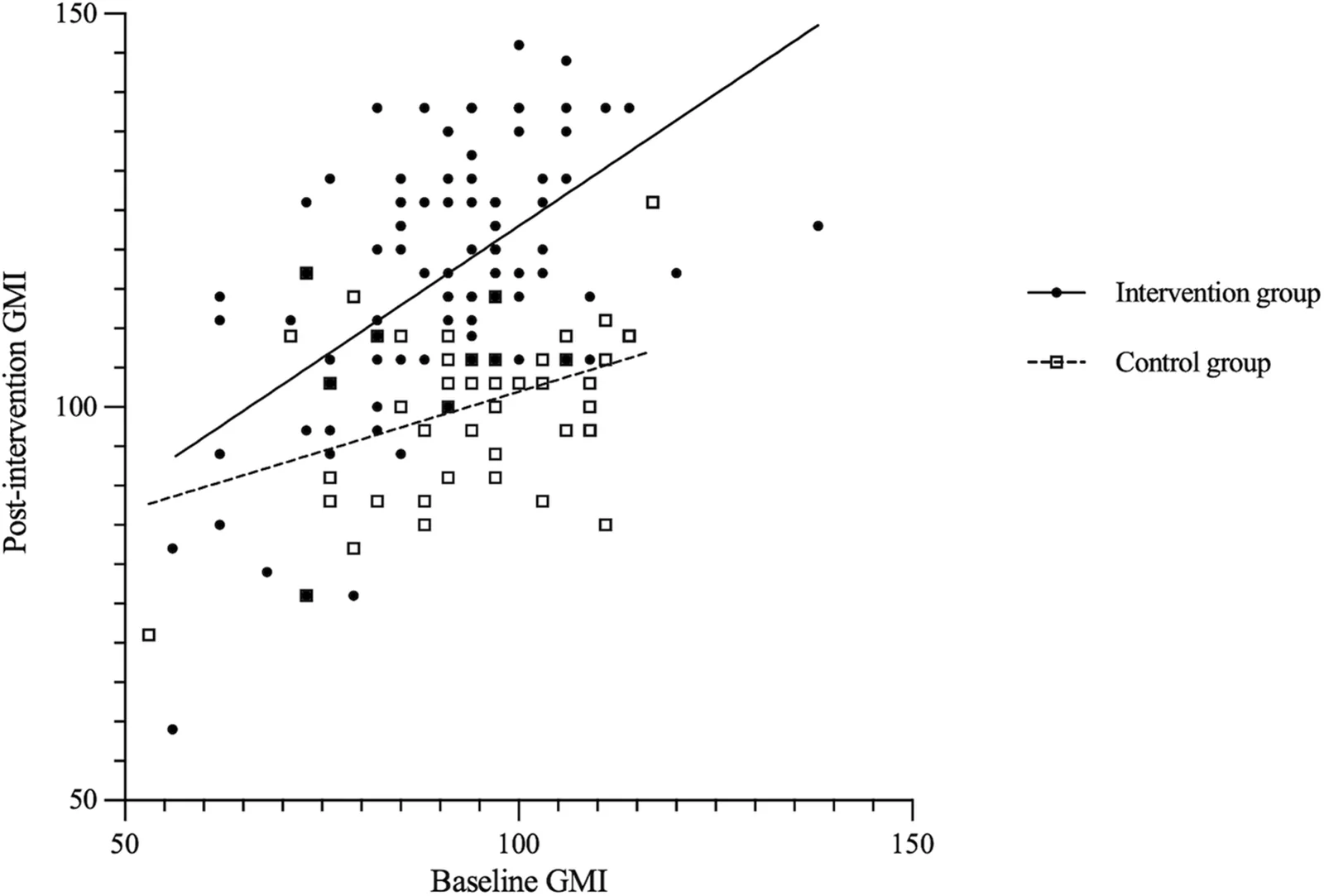
Relationship between baseline and post-intervention Gross Motor Index (GMI) scores in the intervention group (IG) and control group (CG). Regression lines illustrate the association between baseline and post-intervention GMI scores within each group.

Figure 2 shows the relationship between baseline and post-intervention GMI scores separately for the IG and CG. The regression lines indicate different slopes across groups, with a steeper slope in the IG than in the CG, consistent with the observed group × baseline interaction.

Exploratory correlations within the IG are presented in Table 7. Baseline locomotor and ball skills scores were strongly correlated (*r* = .688, *p* < .001). Age was moderately associated with baseline locomotor scores (*r* = .405, *p* < .001), but not with baseline ball skills. Baseline locomotor scores were weakly and negatively associated with change scores (*r* = −.243, *p* = .020), whereas baseline ball skills showed a weak positive association with improvements in ball skills (*r* = .217, *p* = .037). Age was not significantly associated with changes in locomotor skills but showed a moderate positive association with improvements in ball skills (*r* = .521, *p* < .001). Changes in locomotor and ball skills were moderately correlated (*r* = .385, *p* < .001).

**Table 7:** Pearson correlations within the intervention group.

Variable	*r*
Locomotor score (PRE) – Age	.405***
Ball skills score (PRE) – Age	.186
Locomotor score (PRE) – Ball skills score (PRE)	.688***
Δ Locomotor score – Age	.129
Δ Ball skills score – Age	.521***
Δ Locomotor score – Δ Ball skills score	.385***
Locomotor score (PRE) – Δ Locomotor score	−.243*
Ball skills score (PRE) – Δ Ball skills score	.217*

Values are Pearson correlation coefficients (r). Locomotor and ball skills scores refer to raw scores. Change scores (Δ) were calculated as POST−PRE.

PRE, baseline; POST, post-intervention; Δ, change score.

*p < .05.

***p < .001.

## 4 Discussion

The present cRCT examined the effects of a structured play-based motor intervention on FMS among preschool children. Although the intervention was implemented as part of a broader multidisciplinary project targeting health-related behaviors in early childhood, the present paper focused specifically on motor competence outcomes.

The findings indicate that, after adjustment for baseline scores, age, and sex, children exposed to the intervention showed higher post-intervention scores for locomotor skills, ball skills, and GMI than children continuing usual preschool activities. Overall, children in the IG showed greater improvements across all outcomes than those in the CG. These results are consistent with previous systematic reviews and meta-analyses indicating that structured motor skill interventions can improve FMS in early childhood, and object-control skills may be particularly responsive to structured practice (7, 11). They are also consistent with experimental studies reporting improvements in both locomotor and object-control skills following structured physical activity interventions in preschool children (27). The particularly marked improvements observed in ball skills may therefore reflect the greater reliance of object-control skills on guided and task-specific practice opportunities. This interpretation is supported by meta-analytic evidence showing that object-control skills tend to respond particularly well to structured programs (7), likely because they require repeated opportunities to refine complex coordinative patterns that may not be sufficiently stimulated within everyday preschool contexts (14). The parallel improvement in GMI, in turn, likely reflects the contribution of gains in ball skills to the composite index.

Notably, improvements over time were also observed in the CG across all motor competence outcomes. Comparable patterns have been reported in previous preschool studies, where coordination-related outcomes improved in both intervention and comparison groups when comparison children engaged primarily in free-play activities (28), and motor skills also increased over time in both groups despite greater gains in the intervention group (29). Evidence from interventions conducted in older children also suggests that motor competence may improve over relatively short intervention periods, although larger gains are often observed in specific domains among children receiving structured intervention (30). One possible explanation is that motor development during early childhood may be influenced not only by structured interventions, but also by natural maturation and regular exposure to movement opportunities in daily preschool routines. Longitudinal evidence also supports this explanation, showing that higher levels of moderate-to-vigorous physical activity are associated with subsequent improvements in FMS among preschool children (31). Nevertheless, the consistently greater improvements observed in the IG suggest that structured programs may provide additional benefits beyond these general developmental influences.

Intervention effects were not uniform across participants, but varied according to baseline motor competence and age. In particular, larger model-estimated between-group differences were observed among children with higher baseline scores across motor competence outcomes. A similar pattern emerged for age, especially for ball skills and GMI, with older children showing greater post-intervention differences between groups. No comparable age-related moderation was observed for locomotor skills, which may indicate that locomotor tasks were more similarly accessible across the preschool age range, whereas ball skills may be more dependent on age-related coordinative and attentional capacities. Children with higher baseline motor competence may therefore have been better able to engage with and benefit from increasingly complex motor tasks, potentially due to more developed motor repertoires and prior movement experience. Likewise, age-related differences in cognitive, attentional, and coordinative capacities may have facilitated more effective motor learning among older participants. From a developmental perspective, these results are consistent with models proposing that motor competence evolves through cumulative and self-reinforcing processes, whereby baseline motor competence may influence subsequent engagement and responsiveness to practice (32, 33).

To further explore these patterns, Pearson correlations conducted within the IG provided complementary information on individual change patterns. Lower baseline locomotor scores were associated with greater locomotor gains, whereas higher baseline ball skills were associated with greater gains in ball skills. Age was positively associated with improvements in ball skills, but not with changes in locomotor skills, and changes in locomotor and ball skills were moderately correlated. Interestingly, the associations between baseline motor competence and change differed across domains. Although these findings should be interpreted cautiously, they may suggest that locomotor skills are more responsive to intervention even among children with lower baseline motor competence, whereas improvements in ball skills may depend more strongly on pre-existing motor repertoires and prior skill proficiency. Overall, these findings support the notion that the relationship between baseline motor competence and motor growth may be domain-specific, potentially reflecting distinct developmental trajectories and task-specific learning processes, with limited transfer across motor skill categories (4, 34).

In addition to these effects, sex differences were observed for ball skills, with boys showing higher post-intervention scores in ball skills than girls. This finding is consistent with previous literature reporting higher proficiency in object-control skills among boys during childhood, potentially reflecting differences in exposure to ball-related activities, practice opportunities, encouragement, and broader sociocultural influences (35). No sex differences were observed for locomotor skills, suggesting that these differences may be domain-specific rather than generalizable across all components of motor competence. Sex differences were also observed for GMI, likely reflecting the contribution of ball skills to the composite index. However, group × sex interactions were not significant, suggesting that the intervention effects did not differ meaningfully between boys and girls. This reinforces the importance of ensuring equitable opportunities for practice and engagement between boys and girls, particularly in object-control activities, which may be more sensitive to environmental and sociocultural influences.

As part of broader efforts to support early motor competence within physical literacy promotion frameworks, these results support the inclusion of structured play-based motor programs within preschool curricula. In particular, they suggest that early motor competence may benefit from guided, age-appropriate instruction alongside routine free-play opportunities. Repeated practice, appropriate instruction, and instructor feedback may also have supported skill acquisition, in line with evidence emphasizing the role of instructional quality within structured learning environments (13). Similarly, structured active play may be as effective as skill-oriented physical education in improving FMS in preschool children, whereas unstructured active play appears to be less effective (8).

At the same time, the observed moderation effects underscore the importance of adopting flexible and developmentally responsive intervention approaches. Children with higher baseline motor competence and older age appeared to benefit more from the intervention, suggesting that uniform program delivery may not be equally effective for all participants. This highlights the need to adapt task difficulty, instructional support, and feedback to children's individual competence levels, so that less-skilled children remain adequately supported while more advanced children continue to be challenged.

Furthermore, the domain-specific patterns observed across locomotor and ball skills suggest that structured movement programs for preschool children should target a broad range of motor skills, rather than assuming automatic transfer across domains. Providing varied and context-specific movement experiences may therefore help support balanced motor development across skill categories. Consistent with this perspective, recent evidence suggests that improvements in motor competence may differ according to the characteristics of the activities performed and the specific opportunities for practice they provide (36).

Several limitations should be considered when interpreting the present findings. First, although the study adopted a cluster-randomized design, the small number of preschool classes limited the precision with which clustering effects could be estimated, and the initial power calculation did not explicitly account for the cluster-randomized design. However, mixed-effects sensitivity analyses including preschool class as a random intercept yielded results consistent with the primary ANCOVA models. Second, the study was conducted in two public schools within a single urban area in southern Italy, which may limit the generalizability of the findings to other educational settings, geographical areas, or sociocultural contexts. Third, outcome assessors were not blinded to group allocation, which may have introduced potential detection bias, although standardized procedures were implemented to minimize this risk. Additionally, although TGMD–3 is a standardized measure of gross motor development, potential floor or ceiling effects and differences in measurement sensitivity across the preschool age range cannot be entirely excluded and should be considered when interpreting the magnitude of the observed effects.

Attrition represents another limitation, as dropout was higher in the CG than in the IG. Children without post-intervention data showed descriptively higher baseline ball skills scores than completers, although this difference did not reach statistical significance after correction for unequal variances. This pattern could have led to an overestimation of the intervention effect for ball skills in the complete-case analysis, particularly because attrition was higher in the CG. This differential attrition appeared to be mainly related to the timing of post-intervention assessments near the end of the school year, when several children were no longer regularly attending preschool. Nevertheless, multiple imputation sensitivity analyses yielded results consistent with the complete-case findings. Usual preschool activities in the CG were not standardized or quantitatively monitored, limiting the ability to compare the intervention with a precisely defined control condition. Although intervention delivery was supported by predefined session plans, trained instructors, and written attendance records, intervention fidelity was not formally quantified using an independent checklist, which may limit replication and interpretation of implementation consistency. Individual-level socioeconomic variables were not collected, limiting the ability to account for contextual influences on motor development.

Finally, the retrospective trial registration and the absence of follow-up assessments limit conclusions regarding protocol transparency and the long-term maintenance of the observed effects.

In conclusion, the present study found that, in this sample of preschool children aged 3–6 years, a structured play-based motor intervention was associated with greater improvements in FMS and overall motor competence than usual preschool activities. Observed intervention effects varied according to baseline motor competence and, for some outcomes, age, highlighting the importance of considering individual differences in early motor development. These findings suggest that preschool-based structured movement programs may support early motor competence within broader physical literacy promotion frameworks, particularly when activities are adapted to children's developmental level and initial skill proficiency. Future research should examine the long-term sustainability of intervention effects, clarify the mechanisms underlying domain-specific patterns of motor improvement, and investigate how differentiated instructional strategies can be optimized for children with different levels of baseline motor competence.

## Data Availability

The raw data supporting the conclusions of this article will be made available by the authors, without undue reservation.
