# Supplementary material for: Domain-specific motor skill outcomes following a structured play-based motor intervention in preschool children: a cluster-randomized controlled trial
Source: Front Sports Act Living. 2026 Aug 24;8:1917249. doi: 10.3389/fspor.2026.1917249 (PMC13547242; doi:10.3389/fspor.2026.1917249)
Supplement: Supplementary file 1 [file Table1.docx]

| **Supplementary Table S1.** *Comparison of baseline characteristics between completers and dropouts* | | | |
| --- | --- | --- | --- |
| **Variable** | **Completers (n = 139)** | **Dropouts (n = 21)** | **p** |
| **Continuous variables, mean ± SD** |  |  |  |
| Age (years) | 4.70 ± 0.85 | 4.82 ± 0.90 | .562 |
| Weight (kg) | 18.52 ± 3.03 | 17.77 ± 3.11 | .314 |
| Height (m) | 1.07 ± 0.07 | 1.05 ± 0.08 | .345 |
| BMI (kg/m²) | 16.13 ± 1.58 | 15.99 ± 1.15 | .619 |
| Locomotor score | 18.82 ± 9.96 | 16.57 ± 9.66 | .331 |
| Ball skills score | 11.01 ± 3.80 | 13.62 ± 5.89 | .061 |
| GMI | 91.56 ± 13.99 | 89.67 ± 11.96 | .514 |
| **Categorical variables, n (%)** |  |  |  |
| Female | 71 (51.1%) | 11 (52.4%) | .911 |
| Male | 68 (48.9%) | 10 (47.6%) |  |
| Normal weight | 102 (73.4%) | 16 (76.2%) | 1.000 |
| Overweight | 28 (20.1%) | 4 (19.0%) |  |
| Obese | 9 (6.5%) | 1 (4.8%) |  |
| IG | 92 (66.2%) | 6 (28.6%) | <.001 |
| CG | 47 (33.8%) | 15 (71.4%) |  |
| **Note.** Percentages were calculated within completion-status groups. p-values for continuous variables were calculated using Welch’s t-test. p-values for categorical variables were calculated using chi-square tests or Fisher’s exact test, as appropriate. All variables refer to baseline measurements. Locomotor and ball skills scores refer to raw scores.  Abbreviations: BMI, body mass index; CG, control group; GMI, Gross Motor Index; IG, intervention group; SD, standard deviation. | | | |

| **Supplementary Table S2.** *Multiple imputation sensitivity analyses for main intervention effects* | | | |  |
| --- | --- | --- | --- | --- |
| **Outcome** | **Estimate** | **SE** | **95% CI** | **p** |
| Locomotor score | 6.92 | 1.40 | 4.14 to 9.70 | <.001 |
| Ball skills score | 13.05 | 1.44 | 10.20 to 15.91 | <.001 |
| GMI | 17.21 | 2.27 | 12.70 to 21.71 | <.001 |
| **Note.** Values are pooled estimates across 30 imputed datasets. Estimates represent adjusted group differences between the intervention group and control group, with positive values favoring the intervention group. Models were adjusted for the corresponding baseline score, age, and sex. Locomotor and ball skills scores refer to raw scores.  Abbreviations: CI, confidence interval; GMI, Gross Motor Index; SE, standard error. | | | |  |

| **Supplementary Table S3.** *Sex-specific descriptive statistics for ball skills and GMI* | | | |
| --- | --- | --- | --- |
| **Variable** | **Time** | **Female (n = 71)** | **Male (n = 68)** |
| Ball skills score | PRE | 10.41 ± 3.44 | 11.63 ± 4.08 |
| Ball skills score | POST | 21.00 ± 11.11 | 29.01 ± 12.41 |
| GMI | PRE | 89.51 ± 12.74 | 93.71 ± 14.97 |
| GMI | POST | 107.59 ± 16.44 | 114.51 ± 16.22 |
| **Note.** Values are presented as mean ± standard deviation and are based on complete cases. Ball skills scores refer to raw scores.  Abbreviations: GMI, Gross Motor Index; POST, post-intervention; PRE, baseline. | | | |
